# Essential Oils of *Hyptis pectinata* Chemotypes: Isolation, Binary Mixtures and Acute Toxicity on Leaf-Cutting Ants

**DOI:** 10.3390/molecules22040621

**Published:** 2017-04-12

**Authors:** Rosana B. Feitosa-Alcantara, Leandro Bacci, Arie F. Blank, Péricles B. Alves, Indira Morgana de A. Silva, Caroline A. Soares, Taís S. Sampaio, Paulo Cesar de L. Nogueira, Maria de Fátima Arrigoni-Blank

**Affiliations:** 1 Departamento de Engenharia Agronômica, Universidade Federal de Sergipe, São Cristóvão, SE 49100-000, Brazil; rosana.barroso@hotmail.com (R.B.F.-A.); bacci.ufs@gmail.com (L.B.); arie.blank@gmail.com (A.F.B.); indiramorgana@hotmail.com (I.M.d.A.S.); carolalves.10093@gmail.com (C.A.S.); 2 Departamento de Química, Universidade Federal de Sergipe, São Cristóvão, SE 49100-000, Brazil; periclesbalves@gmail.com (P.B.A.); tais.parker@gmail.com (T.S.S.); pclimanog@uol.com.br (P.C.d.L.N.)

**Keywords:** Lamiaceae, pests, secondary metabolites, essential oils profile, major constituents, bio-insecticide

## Abstract

Leaf-cutting ants are pests of great economic importance due to the damage they cause to agricultural and forest crops. The use of organosynthetic insecticides is the main form of control of these insects. In order to develop safer technology, the objective of this work was to evaluate the formicidal activity of the essential oils of two *Hyptis pectinata* genotypes (chemotypes) and their major compounds on the leaf-cutting ants *Acromyrmex balzani* Emery and *Atta sexdens rubropilosa* Forel. Bioassays of exposure pathways (contact and fumigation) and binary mixtures of the major compounds were performed. The major compounds identified in the essential oils of *H. pectinata* were β-caryophyllene, caryophyllene oxide and calamusenone. The essential oils of *H. pectinata* were toxic to the ants in both exposure pathways. Essential oils were more toxic than their major compounds alone. The chemotype calamusenone was more toxic to *A. balzani* in both exposure pathways. *A. sexdens rubropilosa* was more susceptible to the essential oil of the chemotype β-caryophyllene in both exposure pathways. In general, the binary mixtures of the major compounds resulted in additive effect of toxicity. The essential oils of *H. pectinata* is a raw material of great potential for the development of new insecticides.

## 1. Introduction

Cutting-ants of the genera *Atta* and *Acromyrmex* are responsible for high losses in agricultural and forest crops. They are distributed all over the neotropical region [1], and attack plantations throughout the year [2]. The damage is related to the cutting of plant fragments used as substrate for the cultivation of the symbiotic fungus that makes part of cutting-ants’ diet [3].

These insects are able to choose the plant material to be cut, and they may discriminate species of the same genus, and even plants of the same species [4]. This preference for certain plant species is related to the different chemical compositions of the plants [5,6]. Thus, secondary plant metabolites are linked to their susceptibility or tolerance to the attack of leaf-cutting ants. This knowledge allows the use of these compounds in the management of these pests, to the detriment of conventional insecticides [7].

In addition to the economic, social and environmental problems caused by organosynthetic insecticides, the reduced number of recommended active principles, and the requirements of forest certifiers have hindered the management of leaf-cutting ants [8].

The non-governmental organization Forest Stewardship Council (FSC) has set standards in order to implement forest management based on criteria that not only ensure economic viability, but also aggregate social benefits. For this, the certifier has prohibited the use of organosynthetic insecticides in pest control in planted forests [9]. Therefore, efficient and environmentally friendly control methods are essential for the management of leaf-cutting ants. 

Studies have been carried out in the search for bioactive substances as an alternative to organosynthetic insecticides [10,11,12,13,14,15,16,17]. In this context, essential oils of plants appear as an option to control these pests for being efficient and environmentally safer [18,19]. Since essential oils are a mixture of substances of very complex composition, the emergence of populations of resistant insects should slow down, which would consequently increase the time of use of this technology [20]. Usually, the major compound present in the essential oil is responsible for the biological activity. However, in some cases, the combination of these substances may increase the activity of the essential oil, since the compounds may interact with synergistic effect [21,22,23].

Changes in the chemical composition of plants of the same species are due to different environmental pressures (e.g., herbivory and edaphoclimatic conditions) to which they are subjected, and may lead to genetic modification that can result in the formation of chemotypes [24]. The chemotypes, in turn, can provide essential oils with different biological activities [15].

The medicinal and aromatic plant *Hyptis pectinata* (L.) Poit. (Lamiaceae), popularly known in the Brazilian northeast as “sambacaitá”, or “canudinho”, has antidematogenic, antinociceptive [25], antimicrobial [26], insecticide [27], anti-inflammatory [28], and leishmanicidal [29] activities. However, the formicidal activity of the essential oils of this plant has not been studied yet. Thus, results reported in this work enabled the deposit of a patent for the formicidal activity of the essential oils of *H. pectinata* [30].

The objective of this work was to identify and quantify the chemical compounds; to evaluate the toxicity of the essential oils of two *H. pectinata* genotypes (chemotypes) and their major compounds to *A. balzani* and *A. sexdens rubropilosa*; and to determine whether or not synergistic, additism, and/or antagonistic effects occur by testing the binary mixture of the major compounds. Based on the results of the present study, the essential oil of *H. pectinate* and its major compounds β-caryophyllene, caryophyllene oxide and calamusenone showed formicidal activity against *A. balzani* and *A. sexdens rubropilosa,* affecting the survival of these species.

## 2. Results

Table 1 shows the chemical composition of the essential oils of the two chemotypes. Twenty-seven compounds were identified in the essential oils, and 90% of their composition consisted of sesquiterpenes. The genotypes SAM-016 and SAM-019 presented β-caryophyllene (17.66%) and calamusenone (36.08%) as major compounds, respectively. In both chemotypes, the second major compound was caryophyllene oxide (11.52% and 22.89%). Essential oil contents of 0.67% and 0.62% were obtained for the genotypes SAM-016 and SAM-019, respectively.

The essential oils of the two *H. pectinata* chemotypes presented efficient formicidal activity on the species *A. balzani* and *A. sexdens rubropilosa* via contact and fumigation (Table 2 and Table 3).

The doses and concentrations required to kill 50% of ant populations ranged from 3.48 to 8.18 μg/mg, and from 0.59 to 2.15 μg/mg, respectively. The essential oils were more toxic to the ants than their isolated compounds (Table 2 and Table 3). Essential oils of the chemotypes β-caryophyllene and calamusenone were about 1.9 and 3.8 times (*A. balzani*) and 1.3 and 1.3 times (*A. sexdens rubropilosa*) more potent than their respective isolated compounds when applied via contact (Table 2). In the exposure via fumigation, isolated compounds were not efficient in ants control (Table 3). The higher steepness of the curves of lethal dose and concentration of the chemotype calamusenone resulted in low LDs_90_ and LCs_90_, except for *A. sexdens rubropilosa* via contact (Table 2 and Table 3).

Ants’ survival was differently affected when the insects were exposed to essential oils. *A. balzani* was more susceptible to the chemotype calamusenone, and *A. sexdens rubropilosa* was more susceptible to the chemotype β-caryophyllene in both exposure pathways (Table 2 and Table 3). Although the major compounds showed lower toxicity, they maintained the same pattern of that of the essential oils when applied by contact, i.e., calamusenone was more toxic to *A. balzani,* and β-caryophyllene was more toxic to *A. sexdens rubropilosa* (Table 2).

**Figure 1:**
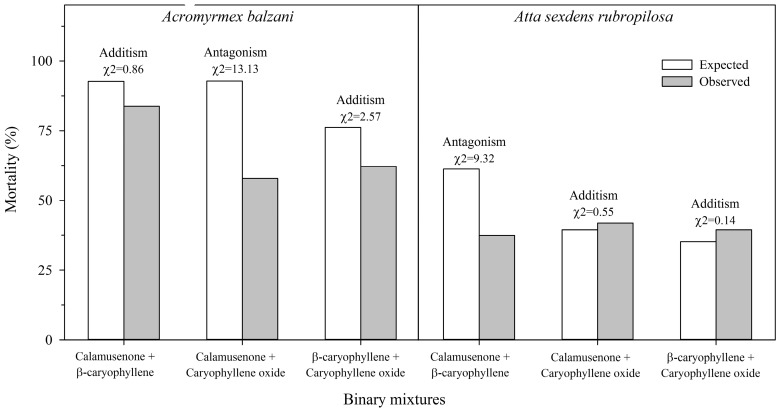
Effect of binary mixtures of the major compounds of the essential oils of two *Hyptis pectinata* chemotypes on the mortality of *Acromyrmex balzani* and *Atta sexdens rubropilosa* at 48 h after exposure via contact.

Most of the time, the binary mixtures of the major compounds of the essential oil of *H. pectinata* showed additive effect, resulting, in some cases, in antagonism between the molecules (Figure 1).

## 3. Discussion

Several studies have reported the chemical diversity among plant genotypes of the same species. The difference in the chemical composition may be related to environmental, genetic and phenological factors [31,32,33]. 

The identification of the compounds present in the essential oils of the genotypes of *H. pectinata* allowed the distinction of these compounds into chemotypes. Studies on the essential oils of this plant have reported variations among their chemical compounds. Different authors have identified β-caryophyllene (12.9%–28.3%), caryophyllene oxide (18.0%–28.0%), calamusenone (24.7%), α-muurolol (25.5%), cubenol (11.4%) and germacrene-D (8.2%) as major compounds in plants collected in the state of Sergipe. In contrast, *H. pectinata* plants from Africa presented *p*-cymene (33.8%) and γ-terpinene (8.9%) as major compounds [34]. The present results corroborate with the diverse chemical composition of essential oils of *H. pectinata* plants, in which β-caryophyllene, caryophyllene oxide and calamusenone have been identified as major compounds [25,26,28].

The essential oils of the two *H. pectinata* chemotypes were toxic to *A. balzani* and *A. sexdens rubropilosa* via contact and fumigation. The comparison with other works allows evaluating the efficacy of these oils. In experiments of toxicity via contact and fumigation with the essential oil of *Aristolochia trilobata* to *Acromyrmex balzani* ants, fumigation presented LC_50_ of 9.33 μL/L. Conversely, the essential oil presented low toxicity via contact [35]. In *Pogostemon cablin*, the concentration required to cause 50% of the mortality of ants of the species *Atta sexdens rubropilosa* by fumigation was of 1.30 μL L^−1^ [36].

The insecticidal activity reported in the present work is a result of bioactive substances synthesized by the secondary metabolism of plants, which can act in the natural defense against the attack of pathogens. Thus, these substances have increasingly been used as sources of raw material in the development of new bio-insecticides, since they have many advantages when compared with synthetic insecticides. The process of insect resistance to these substances is slower and essential oils are biodegradable [37].

The insecticidal activity of the essential oil of this plant has already been demonstrated for larvae of *Aedes aegypti* [27]. Essential oils need to penetrate the body of the insect and reach the site of action, so that they can exhibit their insecticide activity. The present work showed that essential oils can penetrate the ants′ cuticle (contact) or their spiracles (fumigation). The ability of cuticle penetration of the insects can be restricted by the chemical composition of the essential oils, and the physico-chemical properties and thickness of the cuticle [38]. Possibly, the essential oil applied via contact could not penetrate the ants’ cuticle as efficiently as fumigation.

The species *H. pectinata* is popularly and successfully used in the treatment of pain and inflammation. The chemical composition of its essential oils is mainly composed of sesquiterpenes, which are hydrocarbons that have a chemical formula very similar to anti-inflammatory drugs and analgesics, such as fenoprofen and ibuprofen [39]. Anti-inflammatory and nociceptive substances act on the regulation and release of neurotransmitters responsible for inflammatory and pain sensitivity [40,41].

Similarly, once inside the body of the insects, the essential oils possibly act in the nervous system, regulating the neurotransmitters [42,43]. In fact, in the present work, ants presented signs of neurological intoxication, such as spasms, followed by paralysis, and consequent death [44]. Thus, the toxicity of the essential oils or of their major compounds may be due to different mechanisms, such as: (i) octopaminergic sites of action, decreasing or increasing octopamine, a neurotransmitter and neuromodulator found exclusively in invertebrates, may result in the rupture of the nervous system activity, when alterations in the functioning of this neurotransmitter occur [45]; (ii) interference in the chlorine channels modulated by gamma-aminobutyric acid (GABA), a substance that controls the flow of chlorine ions by the nerve cell membrane and restores their resting state, causing hyperexcitation of the nervous system [46]; (iii) inhibition of the acetylcholinesterase enzyme, which catalyzes the hydrolysis of acetylcholine and is a neurotransmitter, causing nervous hyperactivity, and consequent death of the insect [47].

The sesquiterpenes tested in the experiment were less toxic when compared with the essential oils, suggesting the combined participation of the compounds. The biological activity of essential oils is also influenced by interactions between their compounds [48]. The contribution of each compound to the biological activity may depend on the other compounds, as a result of the interaction between the compounds [49]. Thus, the analysis of the chemical composition of the essential oil alone cannot confirm that the major compound is responsible for the biological activity in question, and its effect can be attributed to a compound in a lesser proportion, or to synergism between the compounds [50]. The synergistic effect of the compounds of the essential oils of geranium (*Geranium maculatum* L.) was demonstrated by the insecticidal activity against the domestic fly (*Musca domestica* L.) [51]. Higher insecticidal activity of the essential oil of Thyme (*Thymus vulgaris* L.) and lemon grass (*Cymbopogon citratus* (DC.) Stapf) was confirmed in relation to the major compounds when individually tested [17].

Only exposure via contact allowed determining the curve of the lethal dose when the isolated major compounds were tested. Some compounds of the essential oils may have important participation in the penetration of the cuticle, determining the lipophilic attraction and the cellular distribution [21]. Low toxicity of the major compounds was observed when using the fumigation method. The higher the molecular weight and the boiling point were, the lower the volatility of the chemical compounds [52,53]. β-caryophyllene, caryophyllene oxide and calamusenone are sesquiterpenes, and present high boiling points [54,55,56]. Conversely, the higher the vapor pressure of the compounds, the greater the volatility. Compounds with higher molecular weights have lower vapor pressure [57,58], requiring a longer period for complete volatilization. These characteristics are possibly responsible for the low efficiency obtained via fumigation.

Regardless of the exposure pathway, the essential oils of the chemotypes calamusenone and β-caryophyllene were more efficient in *A. balzani* and *A. sexdens rubropilosa*, respectively. The biological activity of the essential oils is directly related to their chemical compounds and to the physiological/biochemical responses of the insects when exposed to these compounds [48]. These results are due to the differentiated chemical composition of the essential oils studied, as well as to the susceptibility of each ant species to the different compounds present in the essential oil. This susceptibility to different compounds may be related to the enzymatic complex inherent in each ant species, and the consequent capacity to metabolize them, besides the insensitivity of the target site of action [59].

Binary mixtures of major compounds of the essential oil of *H. pectinata* did not result in synergistic effects. Compounds present in smaller proportions in the essential oils can regulate the activity of the major compounds [60]. Toxicity of the essential oil of *H. pectinata* may be related to the synergism between its minor and major compounds, which increases their effectiveness.

Currently, natural plant substances are used as bio-insecticides in family farms, based on traditional knowledge transmitted through generations, and in commercial production. The essential oils of *Azadirachta indica*, *Chenopodium ambrosioides* and *Mentha piperita* are some examples of the raw materials of bio-insecticidal products available in the market [61].

Although plant essential oils have been increasingly tested against a wide range of insects with promising results, the amount of commercial bioinseticides consisting of essential oils is still small. This may be due to insufficient government support, which makes the process of bio-insecticide authorization highly complex and costly. Another factor is the rapid volatilization and oxidation of the chemical compounds of the essential oils, which impairs the chemical stability of the compounds and significantly reduces the persistence of the efficacy in its direct use, requiring the development of efficient stabilization processes, such as formulations [62]. The efficacy of the essential oils of *H. pectinata* used in this work via formicidal formulations has already been proved in patent [30].

The present results demonstrate that the essential oils of *H. pectinata* are a promising alternative to be used in the management of leaf-cutting ants. Thus, the confirmation of the formicidal potential of the essential oils of *H. pectinata* qualifies this species as a source of raw material for formulation and commercialization of bioproducts to control leaf-cutting ants. Additional behavioral studies should be carried out in order to elucidate the behavior of the ants against bioactive substances, since they can reduce the action of these compounds by mechanisms such as chemical communication, olfactory sensitivity and learning ability [63]. At the same time, tests should be carried out to evaluate the activity of these essential oils under environmental conditions.

## 4. Materials and Methods

### 4.1. Plant Material, Extraction and Chemical Analysis of Essential Oils

To obtain the essential oils, leaves of SAM-016 (β-caryophyllene chemotype) and SAM-019 (calamusenone chemotype) of *Hyptis pectinata* (L.) Poit. were collected from the Active Germplasm Bank of Medicinal and Aromatic Plants of the Federal University of Sergipe. The collected leaves were dried in an oven at 40 °C for five days, and subjected to hydrodistillation in a modified Clevenger-type apparatus, for 150 min [64]. The content of the essential oil in the dry leaves (%—expressed in dry leaf mass) was calculated. Voucher specimens were deposited in the herbarium of the Federal University of Sergipe, registration no. 18986 and 18999. 

Analyses of the essential oil compounds were carried out using a GC-MS/FID (QP2010 Ultra, Shimadzu Corporation, Kyoto, Japan), equipped with an autosampler AOC-20i (Shimadzu). Separations were accomplished using an Rtx^®^-5MS Restek fused silica capillary column (5%-diphenyl–95%-dimethyl polysiloxane) of 30 m × 0.25 mm i.d., 0.25μm film thickness, at a constant helium (99.999%) flow rate of 1.2 mL/min. Injection volume of 0.5 μL (5 mg/mL) was employed, with a split ratio of 1:10. The oven temperature was programmed from 50 °C (isothermal for 1.5 min), with an increase of 4 °C/min, to 200 °C, then 10 °C/min to 250 °C, ending with a 5 min isothermal at 250 °C.

The MS and FID data were simultaneously acquired by employing a Detector Splitting System; the split flow ratio was 4:1 (MS:FID). A 0.62 m × 0.15 mm i.d. restrictor tube (capillary column) was used to connect the splitter to the MS detector; a 0.74 m × 0.22 mm i.d. restrictor tube was used to connect the splitter to the FID detector. The MS data (total ion chromatogram, TIC) were obtained in the full scan mode (*m*/*z* of 40–350) at a scan rate of 0.3 scan/s, by using the electron ionization (EI), with electron energy of 70 eV. The injector temperature was 250 °C, and the ion-source temperature was 250 °C. The FID temperature was set to 250 °C, and the gas supplies for the FID were hydrogen, air, and helium at flow rates of 30, 300, and 30 mL/min, respectively. Quantification of each constituent was estimated by FID peak-area normalization (%). Compound concentrations were calculated from the GC peak areas, and they were arranged in order of GC elution. 

Retention indices were determined by the equation of Van den Dool and Kratz (1963) [65], in relation to a homologous series of *n*-alkanes (*n*C9-*n*C18), and compared with retention indices of the literature [66] for the identification of the compounds. Simultaneously, three libraries (WILEY8, NIST107 and NIST21) of the equipment were used, which allowed the comparison of the spectra data with the data of the libraries, using an 80% similarity index.

### 4.2. Obtainment of Major Compounds

**Figure 2:**
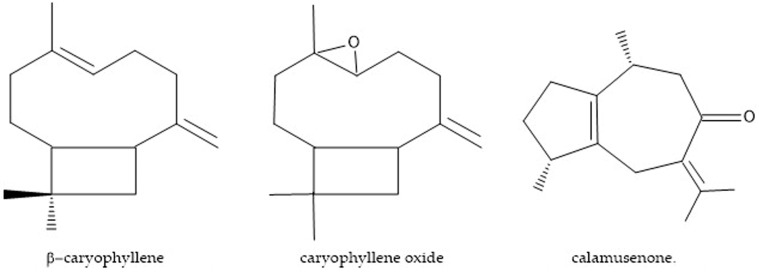
Three major compounds found in the essential oils of two *H. pectinate* chemotypes.

The compounds of the essential oil of *H. pectinata* found in proportions above 11% were considered to be major compounds: β-caryophyllene, caryophyllene oxide and calamusenone. Standards of β-caryophyllene and caryophyllene oxide were purchased from Sigma-Aldrich^®^ (Steinheim, Germany). Calamusenone was isolated and characterized as described by [67] (Figure 2).

### 4.3. Insects

Workers of *A. balzani* and *A. sexdens rubropilosa* were directly obtained from nests from the campus of the Federal University of Sergipe, São Cristóvão-SE, Brazil (10°54′ S, 37°04′ W). The ants were kept in the nest fragments in round plastic containers (50 cm × 20 cm) at ambient conditions (temperature 25–27 °C and relative humidity 60% ± 5%), for 24 h prior to the test, and only distilled water was supplied during this period. Workers of the same size were used in the bioassays.

### 4.4. Bioassays

The bioassays were carried out at the Laboratory of Agricultural Entomology of the Federal University of Sergipe, São Cristóvão-SE, Brazil.

All treatments were diluted in acetone solvent (Panreac-UV-IR-HPLC-GPC PAI-ACS, 99.9% purity). Previous tests have shown that this solvent did not interfere with the survival of workers. The treatments consisted of the essential oil of the chemotypes β-caryophyllene and calamusenone of *H. pectinata* and its major compounds: β-caryophyllene, caryophyllene oxide and calamusenone. Acetone solvent alone was used as control.

#### 4.4.1. Exposure Pathways

The toxicity of essential oils and of their major compounds was evaluated by two exposure pathways: contact and fumigation. The experiment consisted of completely randomized design with four replications.

Preliminary bioassays were carried out with three doses (0.1; 1.0; and 10 μg essential oil/mg insect) for contact, and three concentrations (0.1; 1.0; and 10 μL essential oil/L air) for fumigation. From these tests, doses and concentrations for the subsequent bioassays were determined in order to obtain the curves of lethal dose and concentration. For the calculation of the doses, the mean weight of 30 ants was obtained, using a 0.01 mg analytical precision scale (Shimadzu AUW220D, Kyoto, Japan)

In the contact bioassays, tests were carried out on Petri dishes (9 cm in diameter × 2 cm in height), with filter paper at the bottom (Unifil, code 501.009), moistened with 0.5 mL distilled water, with seven ants per dish. Preliminary tests indicated that this combination of moisture and ant density does not affect ants’ survival. Petri dishes were kept in a freezer at −4°C for 1 min to reduce ant activity and allow topical application of the treatments to the individuals. Each ant received 1 μL of the essential oils of *H. pectinata* or of the major compounds in the pronotum region, applied using a 10 μL Hamilton^®^ microsyringe. The dishes containing the ants were sealed with PVC plastic film and kept in B.O.D. chamber at 25 ± 1 °C and relative humidity > 70%.

In the fumigation bioassays, glass vials (250 mL) with filter paper at the bottom were moistened with 0.5 mL distilled water containing seven ants per vial. Preliminary tests indicated that this combination of moisture and ant density does not affect ants survival. Treatments containing the essential oils or major compounds were applied with the aid of a 10 μL Hamilton^®^ microspheres on 1 cm^2^ filter paper (volatile compound dispersant), fixed by a cotton line at the bottom of the bottle cap.

The filter paper was kept in the center of the bottle, out of reach of the ants, and avoiding direct contact with the insect. The vials were hermetically sealed with PVC plastic film and plastic cap, and kept in B.O.D. chamber at 25 ± 1 °C, and relative humidity >70%.

Mortality was evaluated 48 h after the start of the bioassay. Individuals that failed to move after prodding with a brush were considered dead.

#### 4.4.2. Binary Mixtures

The effects of the binary mixtures of the major compounds (β-caryophyllene, caryophyllene oxide and calamusenone) were determined by a methodology similar to that used in the contact bioassay (2.4.1). The ants were not exposed to binary mixtures via fumigation due to the low toxicity of these compounds in this exposure pathway.

The LD_50_ values of the most effective major compounds determined in the contact bioassay were used in the bioassays. The compounds were grouped in pairs using doses at 1:1 ratio. The major compounds were tested separately and in mixtures at these doses for each ant species. Actual mortality (observed) was compared with expected mortality, and the effects of binary mixtures were classified as additism (or no effect), synergism or antagonism.

### 4.5. Statistical Analyses

Results of mortality from the bioessays of the exposure pathways and the effects of binary mixtures were corrected for the mortality that occurred in the control using the Abbott’s formula [68].

Probit analyses were carried out to determine the curves of lethal dose and concentration of the essential oils of the two *H. pectinata* chemotypes and the major compounds for each ant species. Curves with probability (*p* < 0.05) of acceptance of the null hypothesis were accepted by the χ^2^ test. Lethal doses (LD_50_ and LD_90_) and lethal concentrations (LC_50_ and LC_90_), and their respective confidence intervals at 95% probability (CI_95_) were obtained in the SAS software (version 9.1, Cary, NC, USA), by using these curves [69].

In the bioassays of the binary mixtures, the expected mortalities were calculated according to the formula described by [70]: *E* = *O*a + *O*b (1 − *O*a), where *E* is the expected mortality and *O*a and *O*b are the observed mortalities caused by the major compounds separately.

The effects of the binary mixtures were classified by comparing the calculated χ^2^ and the table *χ*^2^ (*χ*^2^_tab_ = 3.84; df = 1; α = 0.05). *χ*^2^ was calculated using the following formula:

$$\chi^{2}=\frac{{\left(Om-E\right)}^{2}}{E}$$

where *O*m is the observed mortality of the binary mixture.

The value of the analyzed pair with *χ*^2^_cal_ < 3.84 indicates that the effect is additism (no effect). Values of the analyzed pair with *χ*^2^_cal_ > 3.84 indicate that the effect is either synergism or antagonism. In this last case, the expected and observed mortalities of the binary mixtures should be observed.

## Figures and Tables

**Table 1:** Chemical composition of the essential oils of two *Hyptis pectinata* chemotypes.

Compound	RRI ^a^	Concentration (%) ^b^ of the Compounds in *H. pectinate* Chemotypes	
		Plant SAM-016	Plant SAM-019
β-pinene	974	2.26	-
*p*-cymene	1020	1.32	-
Limonene	1024	1.65	-
δ-elemene	1335	3.92	-
α-cubenene	1345	1.56	0.31
α-copaene	1374	4.85	1.12
β-bourbonene	1387	1.02	0.47
β-elemene	1389	7.79	2.05
β-caryophyllene	1417	17.66	9.42
γ-elemene	1434	-	0.63
(*Z*)-muurola-3,5-diene	1448	-	0.56
α-humulene	1452	1.66	0.81
γ-muurolene	1478	5.66	-
Germacrene D	1484	-	1.87
(*Z*)-β-guaiene	1492	-	4.77
Bicyclogermacrene	1500	4.57	-
Lepidozene	1502	-	0.68
γ-cadinene	1513	-	2.72
(*E*)-calamenene	1521	5.76	1.81
Spathulenol	1577	10.09	-
Caryophyllene oxide	1582	11.52	22.89
Globulol	1590	1.82	-
α-acorenol	1632	-	0.15
Epi-α-cadinol	1638	4.88	-
Cubenol	1645	-	3.29
α-cadinol	1652	2.22	1.59
Calamusenone	1676	-	36.08
Essential oil content (%)		0.67	0.62

^a^ RRI = relative retention index. ^b^ The concentration (%) of each compound represents the mean of three replications, and the traces indicate that the compound was not detected in these essential oils.

**Table 2:** Toxicity via contact of the essential oils of two *Hyptis pectinata* chemotypes and their major compounds to two species of leaf-cutting ants.

Treatment	N. Insects	LD_50_^a^ (95%CI) (μg mg^−1^)	LD_90_ (95%CI) (μg mg^−1^)	Slope ^b^	Χ^2^	*p*-Value
*Acromyrmex balzani*						
Chemotype β-caryophyllene	391	8.18 (7.09–8.96)	31.16 (22.17–52.47)	2.20	2.59	0.53
Chemotype calamusenone	406	3.48 (3.05–3.91)	9.84 (8.32–12.42)	2.84	2.97	0.22
β-caryophyllene	224	15.59 (13.87–17.68)	47.31 (37.41–65.98)	2.66	1.92	0.62
Calamusenone	811	13.21 (12.56–13.82)	18.93 (17.87–20.40)	8.19	1.90	0.61
Caryophyllene oxide	252	18.97 (17.00–21.08)	49.53 (40.47–66.70)	3.05	2.97	0.22
*Atta sexdens rubropilosa*						
Chemotype β-caryophyllene	310	3.61 (3.13–4.20)	11.67 (9.20–16.09)	2.51	2.01	0.36
Chemotype calamusenone	406	4.65 (3.77–5.52)	25.80 (18.05–47.17)	1.72	3.60	0.16
β-caryophyllene	392	4.76 (4.14–5.46)	14.74 (12.09–18.99)	2.60	0.24	0.88
Calamusenone	335	5.96 (5.25–6.61)	12.19 (10.88–14.11)	4.12	5.18	0.07
Caryophyllene oxide	644	35.34 (29.47–40.99)	158.92 (107.62–355.32)	1.96	0.05	0.97

^a^ LD_50_—Lethal Dose; ^b^ Slope of the curve.

**Table 3:** Toxicity via fumigation of the essential oils of two *Hyptis pectinata* chemotypes and its major compounds to two species of leaf-cutting ants.

Treatment	N. Insects	LC_50_^a^ (95%CI) (μL L^−1^)	LC_90_ (95%CI) (μL L^−1^)	Slope ^b^	Χ^2^	*p*-Value
*Acromyrmex balzani*						
Chemotype β-caryophyllene	294	1.82 (1.57–2.08)	6.34 (5.14–8.37)	2.36	1.86	0.60
Chemotype calamusenone	173	0.59 (0.53–0.65)	1.35 (1.17–1.63)	3.58	4.50	0.10
β-caryophyllene	497	>100.00 *	-	-	-	-
Calamusenone	441	>100.00 *	-	-	-	-
Caryophyllene oxide	495	>100.00 *	-	-	-	-
*Atta sexdens rubropilosa*						
Chemotype β-caryophyllene	203	1.18 (0.95–1.41)	6.15 (4.47–10.40)	1.79	5.30	0.06
Chemotype calamusenone	171	2.15 (2.04–2.29)	3.52 (3.18–4.05)	6.01	1.52	0.52
β-caryophyllene	422	>100.00 *	-	-	-	-
Calamusenone	392	>100.00 *	-	-	-	-
Caryophyllene oxide	392	>100.00 *	-	-	-	-

^a^ LC_50_- Lethal Concentration; ^b^ Slope of the curve. * Lethal Concentration curves could not be traced due to the low toxicity of the compound.
